# Effects of Seed Colour and Regulated Temperature on the Germination of *Boswellia pirottae* Chiov.: An Endemic Gum- and Resin-Bearing Species

**DOI:** 10.3390/plants13243581

**Published:** 2024-12-22

**Authors:** Shiferaw Alem, Lukáš Karas, Hana Habrová

**Affiliations:** Department of Forest Botany, Dendrology and Geobiocoenology, Faculty of Forestry and Wood Technology, Mendel University in Brno, Zemědělská 1, 613 00 Brno, Czech Republic; xshifera@mendelu.cz (S.A.); lukas.karas@mendelu.cz (L.K.)

**Keywords:** seed colour, seed weight, genetic resources, threatened species, frankincense

## Abstract

(1) Background: According to the IUCN, *Boswellia pirottae* is classified as a vulnerable species. However, knowledge of its seed characteristics and germination behaviour is lacking. (2) Methods: The aim of this research was to characterise the seeds and evaluate the effects of seed colour and controlled temperatures on seed germination. The seeds were segregated into the following colour categories: light brown (LB), brown (B), and dark brown (DB). The seeds were evaluated under controlled constant temperatures (23 °C) and at room (fluctuating) temperature independently. One-way ANOVA, *t*-test, and germination indexes were used for analyses. (3) Results: The results showed significant differences in the mean seed masses of LB, B, and DB seeds. Similarly, the differently coloured seeds varied in their water imbibition rates. The result showed significant differences in the mean germination of the seeds in both the controlled temperature (23 °C) and room-temperature chambers among the LB, B, and DB seeds. However, the *t*-test revealed no significant differences in the mean germination of the seeds of similar colours between controlled temperature and room temperature conditions. (4) Conclusions: The seed’s colour significantly influenced the seed mass, water imbibition capacity, and germination rate relative to the temperature treatment. Dark brown seeds are recommended for seed collection aimed at seedling propagation.

## 1. Introduction

Several studies have documented that frankincense species worldwide face various challenges, such as habitat loss, mortality, limited regeneration, and pest infestation [1,2,3,4,5,6,7]. It is also documented that the germination rates of most *Boswellia* Roxb. ex Colebr. (Burseraceae) species are low, which is attributed to the significant presence of empty or insect-damaged seeds and other factors, such as the tapping intensity, storage conditions, seed collection site, or seed treatment [8,9,10]. Eslamieh [11] indicated that the germination trials performed on 10 *Boswellia* species from Africa and India exhibited relatively poor germination rates that reached 15–30%. Studies showed that the germination rates of the Burseraceae species are notably low, with six *Bursera* species from Mexico ranging from 0% to 18% [12,13]. Similarly, the mean germination rates of *Commiphora wightii* (Arn.) Bhandari, *Canarium resiniferum* Bruce ex King, and *Protium serratum* (Wall. ex Colebr.) Engl were 8–30% [14,15]. Pre-sowing treatments also influenced the germination of *Boswellia sacra* Flück., a crucial frankincense source from southern Arabia and Somalia, and it displayed germination rates ranging from 8% to 69% [10,11]. Seed collection sites influenced the germination of some *Boswellia* species from Socotra [9]. For example, depending on the seed collection locality, the germination rate of *Boswellia ameero* Balf.fil. ranged from 3% to 60%; that of *Boswellia bullata* Thulin ranged from 1% to 61%; that of *Boswellia dioscoridis* Thulin ranged from 3% to 80%; and that of *Boswellia nana* Hepper ranged from 42% to 47% [9]. Over-tapping is another factor negatively affecting seed germination; in the case of *B. papyrifera* (Delile) Hochst., seeds from untapped trees have considerably higher germination rates than seeds from tapped trees (94% and 16%, respectively) [8].

Various factors, including a combination of seed characteristics and environmental conditions, influence the germination of seeds [16]. The environmental factors affecting seed germination include temperature, light, pH, soil moisture, oxygen, and nutrients, while the internal factors include vitality, viability, and seed dormancy [17,18,19]. Plants have developed light receptors called phytochromes, which play an important role in detecting the position of the seed and regulating germination inhibitors that are associated with photoperiods [20,21]. The pH levels also impact germination, with inhibition observed at levels lower than 3 and higher than 8, while most plants germinate optimally within the pH range of 5.5–7 [22,23]. Temperature plays a crucial role in seed germination [24,25]. It influences the rate of germination, which, in turn, governs water absorption and both biochemical and physiological metabolic processes [26]. Plant species exhibit diverse optimal temperature ranges for seed germination, reflecting their adaptation to native habitats [27,28]. Optimal temperatures foster seed germination and promote robust early seedling growth [26,29,30,31]. For most plants, the optimal and maximum germination temperatures range between 15–30 °C and 30–40 °C, respectively [32,33]. Different plant species may prefer either fluctuating or constant temperatures, and temperatures ranging from 20 °C to 35 °C are conducive to the germination of most tropical plant seeds [19,21,25,29,30,34,35,36,37]. Many plants in colder areas require cold stratification (0–10 °C) to break dormancy [38]. However, these temperatures may have adverse effects on the seeds of different species from tropical areas [39]. Lower temperatures could slow the metabolism of these seeds, which results in a delay in seed germination [19]. Temperatures above 40 °C hinder seed germination by inhibiting protein and nucleic acid synthesis, preventing radicle and shoot elongation [21,40].

Morphological heteromorphy of the seeds, referring to variations in the seeds’ size, shape, and colour [41], can also have a substantial impact on their physiological properties, such as dormancy, germination, and longevity [42]. The maternal genotype affects seed development as well, determining the specific pigment deposited within seed cells, with colour acting as an indicator of maturity and potentially influencing seed germination [43,44,45]. Seed coat colour influences the seed by affecting its water uptake, gas diffusion, dormancy, seed quality, and light-filtering properties [46,47,48,49,50,51]. Different research studies have shown that dark-coloured seeds often exhibit higher germination rates than light-coloured ones, with differences observed in their water absorption rates and seed mass [14,49]. *Commiphora wightii* is yet another member of the Burseraceae family with documented differences in seed colours, such as black, brown, and white seeds. Among these, black seeds have the highest germination rate [14,52]. In contrast, in other species, such as *Lotononis platycarpa* (Viv.) Pic.Serm. (Fabaceae), orange seeds showed significantly higher germination rates than those with olive green and brown seed colours [53].

*Boswellia pirottae* Chiov., a member of the Burseraceae family, which is known for its gum and resin production, is an endemic species to Ethiopia. The species is found in the dry tropical biome and distributed in the northwest and western central regions of Ethiopia [54]. *B. pirottae* populations are isolated and restricted to a few areas (Tekeze, Abay, and Gibe River systems), and they grow at altitudes of 1200–1800 m a.s.l. The conservation status of the species is concerning, as it is classified as vulnerable under criterion C1 by the IUCN Red List of Threatened Species due to habitat degradation associated with human activities and population increase [55,56]. This species is highly valued for the production of oleo-gum frankincense, which has historical use in various cultural practices, including traditional medicine, coffee ceremony, cosmetics, and pharmacology [57]. Rural communities in Ethiopia rely on the harvesting of frankincense from this species for both economic and traditional medicinal purposes [58].

Despite the economic and environmental significance of *B. pirottae*, knowledge of the seed and basic germination characteristics is lacking. Moreover, we observed that there is variation in the seed colour, but how it affects its germination has not yet been investigated. Understanding how seed colour and temperature affect seed germination in *B. pirottae* species will be helpful for quality seedling production, afforestation, and genetic conservation purposes. Hence, the objectives of the study were to (1) evaluate the effect of seed colour on the mean seed mass, water imbibition, and mean germination of *B. pirottae* seeds and (2) investigate how controlled temperatures influence the germination process of *B. pirottae* seeds relative to uncontrolled room temperatures. Initially, it was hypothesised that (1) there is variation in the mean seed mass and water imbibition capacity of the different coloured seeds of *B. pirottae,* and (2) controlled temperatures would not significantly impact the germination of the seeds of *B. pirottae* relative to uncontrolled room temperatures.

## 2. Results

### 2.1. Seed Characteristics and Water Imbibition

**Figure 1:**
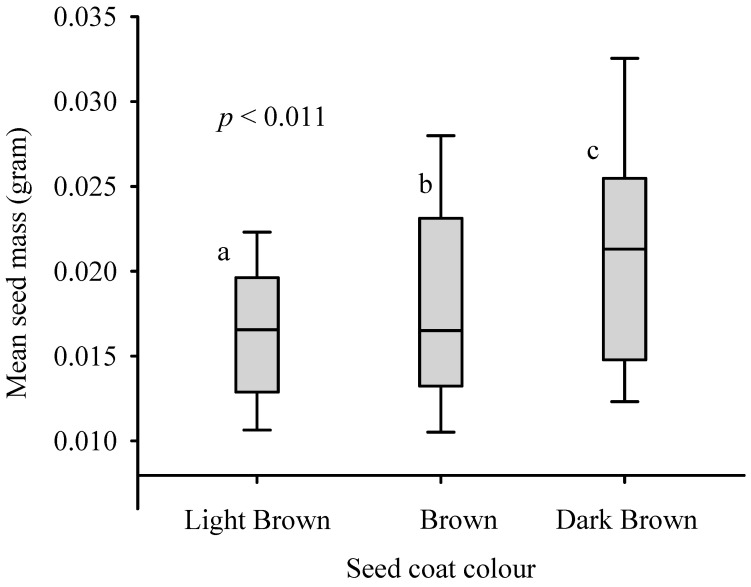
One-way ANOVA results showing the mean mass of *B. pirottae* seeds with different colours. Means with different letters are significantly different from each other at the 0.05 significance level.

The mean number of seeds per fruit ranged between one and four. Approximately 5%, 33%, 53%, and 9% of the fruits had one, two, three, and four seeds, respectively. The result showed that *B. pirottae* has about 59,880 seeds per kg. The mean seed masses of the differently coloured seeds of *B. pirottae* with respect to the LB, B, and DB seeds were 0.016 g, 0.019 g, and 0.021 g, respectively. A one-way ANOVA revealed significant differences in the mean seed mass among different seed colours (*p* = 0.001, Figure 1).

**Figure 2:**
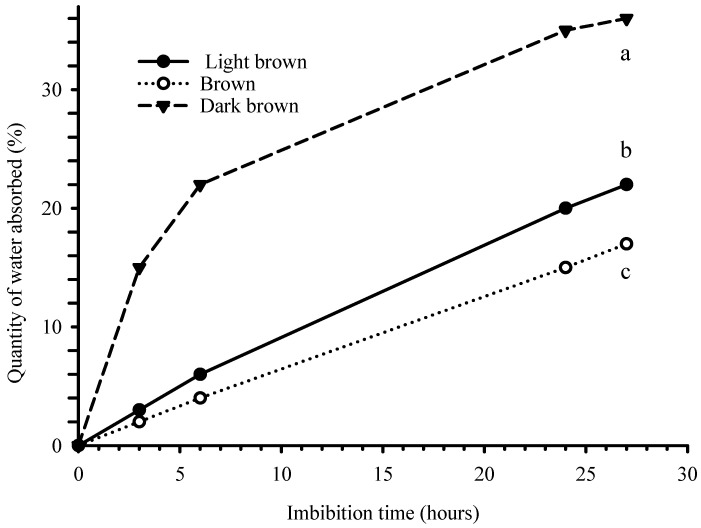
Water imbibition rate of differently coloured *B. pirottae* seeds over time. The letters at the 27 h mark indicate the results of a one-way ANOVA test at a significance level of *p* = 0.05. Different letters represent statistically significant differences.

**Figure 3:**
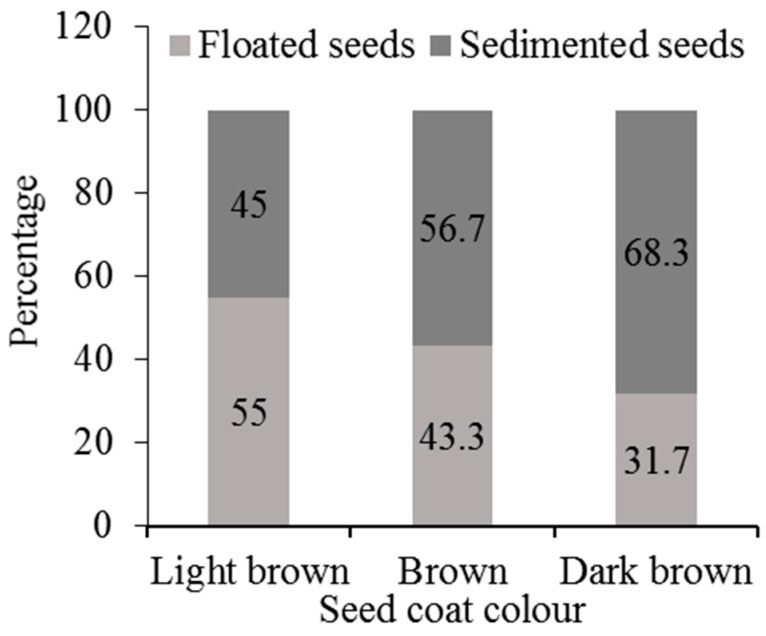
Mean percentage of seeds that floated and settled (sedimented) for the light brown, brown, and dark brown seeds.

The mean water imbibition rates of the seed colour categories are presented in Figure 2. The results showed that the mean water imbibition rate of DB seeds was higher than in B and LB seeds. The one-way ANOVA result exhibited significant differences in the mean water imbibition rates among the differently coloured seeds after 27 h of soaking (*p* < 0.05, Figure 2). The mean percentage of floating seeds was the highest for LB seeds (55%), followed by B seeds (43.3%), and then DB seeds (31.7%) (Figure 3). However, the one-way ANOVA result revealed no significant differences in the mean percentage of floating seeds among the different seed colours of the species (*p* = 0.139).

### 2.2. Effect of Seed Colour and Temperature on Germination

**Figure 4:**
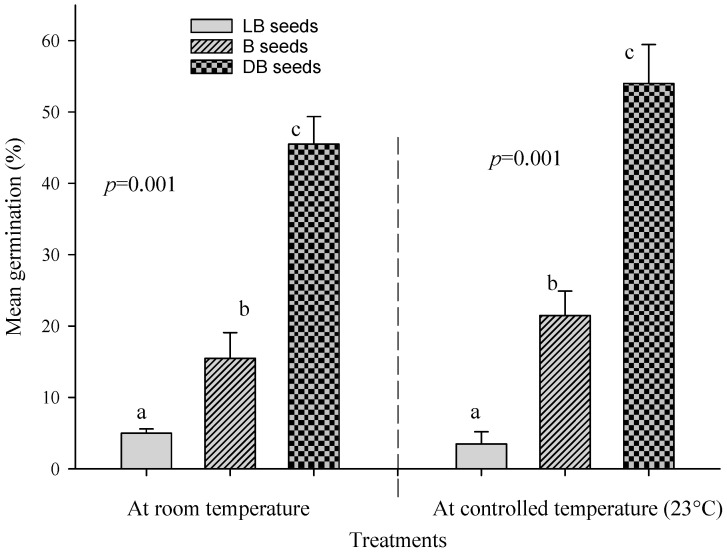
One-way ANOVA results of the mean germination percentage of differently coloured seeds at controlled and room temperatures. Identical letters indicate no significant differences at a *p*-value of 0.05.

The mean germination percentages of LB, B, and DB seeds of *B. pirottae* at room temperature were 5%, 15%, and 45%, respectively. In contrast, the mean germination percentages at a controlled constant temperature (at 23 °C) for LB-, B-, and DB-coloured seeds were 3.5%, 21.5%, and 54%, respectively. The one-way ANOVA result showed significant differences in the mean germination percentages among LB, B, and DB seeds germinated at room temperature (*p* = 0.001, Figure 4). Similarly, the one-way ANOVA result revealed significant differences in the mean germination percentages of seeds germinated at a controlled constant temperature (23 °C) (*p* = 0.001, Figure 4). The mean germination times for LB, B, and DB seeds at room temperature were 9.6 days, 8.2 days, and 8.9 days, respectively, while the mean germination times for LB, B, and DB seeds at the constant controlled temperature (23 °C) were 5.3 days, 7.9 days, and 8.4 days, respectively. The mean germination indices for LB, B, and DB seeds at room temperature were 7.5, 28.3, and 78.9, respectively. In contrast, at a controlled constant temperature (23 °C), the mean germination indices for LB, B, and DB seeds were 12.3, 68.5, and 166, respectively.

**Figure 5:**
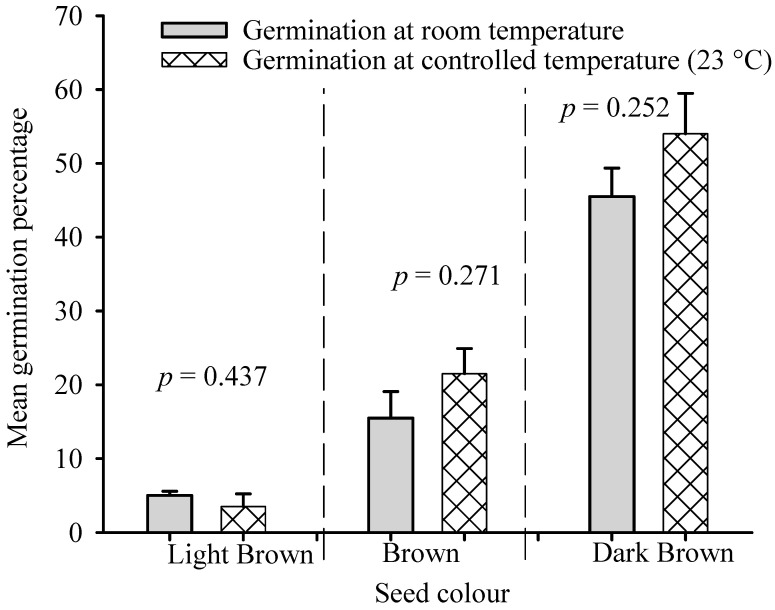
Statistical *t*-test results comparing the mean germination percentages of seeds with similar colours under controlled and room temperatures at a significance level of *p* = 0.05.

**Figure 6:**
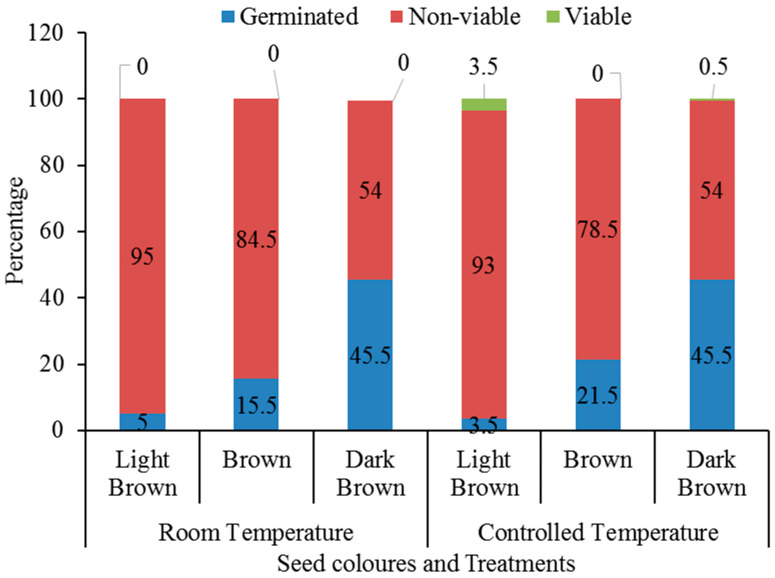
Mean percentage of germinated, viable, and non-viable seeds for different seed colours at the end of the experiment under room temperature and controlled temperature conditions.

**Figure 7:**
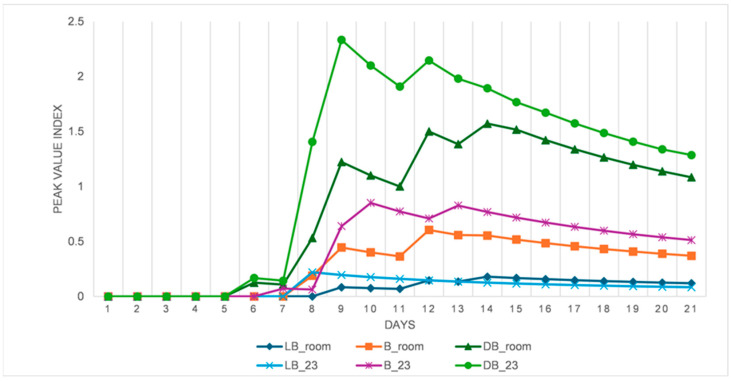
Peak value indices of *B. pirottae* seeds germinated at room (fluctuating) temperature and in controlled conditions at a constant 23 °C (LB—light brown seeds; B—brown seeds; DB—dark brown seeds; and room—room temperature; 23—constant 23 °C).

The statistical *t*-test analysis results on the effects of controlled temperatures on the mean germination of *B. pirottae* seeds relative to room temperature for differently coloured seeds are presented in Figure 5. The analysis results showed no significant differences between the mean germination percentage of LB seeds evaluated at the uncontrolled room temperature and the seeds germinated at the controlled constant temperature (23 °C) (*p* = 0.437, Figure 5). Similarly, there were no significant differences between the mean germination percentages of B-coloured seeds germinated at the controlled constant temperature (23 °C) and the mean germination of seeds at room temperature (*p* = 0.271, Figure 5). Likewise, the *t*-test result revealed no significant differences between the mean germination percentages of DB seeds germinated at room temperature and the seeds germinated at the controlled constant temperature (23 °C) (*p* = 0.252, Figure 5). The viability test result indicated that the proportion of non-viable seeds exceeded the number of germinated and viable seeds for all seed colours (Figure 6). The germination course showed differences when room temperature conditions and controlled conditions of 23 °C were compared (Figure 7). A higher daily germination mean value was observed in seed lots with respect to controlled and constant temperature conditions (23 °C), while in room temperature conditions, the peak values were always lower (Figure 7). Seeds started germinating on the sixth day of the germination trial, with the highest peak values observed in the second week of the trial (Figure 7).

## 3. Discussion

The results of this study indicate that the mean germination rate of *B. pirottae* is 24%, which corresponds to the results of Eslamieh (20%) [11]. The highest germination rate was recorded for dark brown seeds at controlled temperatures (54%), followed by dark brown seeds at room temperature (45%). On the other hand, light brown seeds reached the lowest germination rate in both conditions (5% and 3.5%). Unfortunately, seed colour variations have not yet been analysed in other species of the genus *Boswellia*.

The results also show that both controlled (23 °C) and uncontrolled room temperatures (mean temperature 21 °C) are suitable for the germination of *B. pirottae* seeds, and the results did not show statistically significant differences with respect to germination. The mean germination rates between similarly coloured seeds did not result in statistically significant differences between controlled conditions within a germination chamber maintained at a constant temperature of 23 °C and fluctuating room temperature conditions. This result could suggest that *B. pirottae* is capable of germinating effectively under both constant (23 °C) and fluctuating temperatures ranging from 19 to 25 °C. Similar results were achieved in other tropical species such as *Aspidosperma tomentosum* Mart. [25], *Diospyros blancoi* A.DC. [37], *Tabebuia ochracea* A.H. Gentry [34], and *Tibouchina mutabilis* (Vell.) Cogn. [29]; the results showed that these species can germinate in both constant and fluctuating temperatures. Zamith et al. [36] also indicated that *Melocactus violaceus* Pfeiff. preferred fluctuating temperatures within the range of 20–35 °C. On the other hand, studies showed that *Diptychandra aurantiaca* Tul. exhibited a preference for a constant temperature of 25 °C [35].

Seed heterogeneity across different species is caused by a combination of physiological, environmental, and genetic factors, according to different studies [50,59,60]. For instance, an investigation on *Dalbergia granadillo* Pittier revealed the presence of seeds in two distinct colours: dark brown and light brown [61]. Similarly, *Lotus glinoides* Delile produces seeds that have black and yellow colours, while *Lotus halophilus* Boiss. and Spruner has green and yellow seeds [62]. Accordingly, our current study on *B. pirottae* revealed variations in seed colour regardless of whether seeds were collected within a single day or were collected from different trees within the species’ habitat. Additionally, differences in seed masses among the various coloured seeds were observed, with a general trend indicating higher mean masses for dark brown (DB) seeds followed by brown (B) and then light brown (LB) seeds. These differences in seed colour may be associated with (a) variances in the seed’s physiological ripening period, even though mature seeds were collected, and/or (b) genetic factors governing the species’ adaptation mechanisms to cope with the frequent fires that occur in its habitat. *B. pirottae* grows in a fire-prone area, and studies have shown that seed colour variations could be a survival strategy that determines the time of germination throughout the year [63]. Some studies also showed that seed colour also influences seed dormancy and germination processes [47,62], and others indicated that unripe seeds contain high levels of active gibberellic acid impeding germination [63,64]. Moreover, the lower light brown seed germination rate of *B. pirottae* could be associated with seed mass, as it was lower than the mass of dark brown and brown seeds. This is further supported by the observed differences in the mean seed mass among various seed colours, indicating the species’ potential variations in physiological developmental stages and seed quality. Seed size is an important aspect of seed biology and represents the “investment” made by the parent plant relative to the success of individual seeds in producing seedlings [65].

Various studies have reported variations in the mean germination percentages among seeds of different colours, which is consistent with our study’s findings. A study result of Cruz-García et al. [61] indicated higher germination rates (96%) in the light-coloured seeds of *Dalbergia granadillo*. Similarly, Bhatt et al. [62] found significantly lower mean germination in yellow-coloured seeds compared to the black-coloured seeds of *Lotus glinoides*. Vidak et al. [66] also found that the white-coloured seeds of *Phaseolus vulgaris* L. germinated faster than dark-coloured seeds. Seed colour can influence water absorption rates, thereby impacting seed germination, and this could be the cause for the mean germination variation of the differently coloured seeds of *B. pirottae*. In the current study on *B. pirottae*, dark brown seeds exhibited a relatively higher water imbibition rate compared to brown and light brown seeds, which could potentially influence germination. Some research findings have shown that dark-coloured seeds absorb water more rapidly and consequently exhibit higher germination rates compared to light-coloured seeds [44,48]. Liu et al. [48] observed rapid water uptake and higher germination rates in the black seeds of *Cyamopsis tetragonoloba* (L.) Taub. compared to white seeds. It is indicated that the colours of the structures surrounding the seed may affect light-filtering properties, thus influencing germination requirements, especially the required light during incubation [62,67].

Variability in seed mass is common both within and among plant species [68]. The current study result showed variations in the mean seed masses of the differently coloured seeds (DB > B > LB) of *B. pirottae*. The mean thousand seed mass of *B. pirottae* (across all colour variations) averaged 18.68 g (16.35 g for LB, 18.75 g for B, and 20.93 g for DB). This result is comparable to the thousand seed mass of *B. papyrifera* (15.8 g) [69], and it is greater than *B. sacra* seeds (5.5–6.7 g) [10]; this is possibly due to the larger dimensions of *B. papyrifera* and *B. pirottae* seeds. The seed mass variations in our study could influence seed germination, potentially linking to differences in the food reserves of cotyledons. Research implies that seed germination is largely influenced by the food reserves within seeds, which generally increase with seed mass [70]. Heavy seeds have been shown to germinate earlier and exhibit better germination rates than small seeds under laboratory and greenhouse conditions, as observed in *Prunus jenkinsii* Hook. f. and Thomson [71]. Additionally, within a species, heavier seeds may germinate more quickly than lighter seeds [72]. However, some studies have reported contrary findings, suggesting that lighter seeds may germinate earlier than heavier ones [73]. Perez-Garcia et al. [74] showed that the germination time of a species is independent of its seed mass. For *B. pirottae*, the variation in seed mass, coupled with seed colour differences, may contribute to variations in the mean germination time and mean germination percentage observed among the different-coloured seeds of the species.

## 4. Materials and Methods

### 4.1. Seed Collection and Preparation

In March 2022, only mature fruits of *B. pirottae* from 50 different trees were gathered in the Gibe Valley, located approximately 180 km southwest of Addis Ababa, Ethiopia. The collected fruits were carefully transported to Addis Ababa, mixed, and allowed to dry under shade. The dried seeds were carefully separated from the fruits. The seeds were cleaned, packaged in a plastic container, and stored in a seed storage room that had a temperature of 5 °C until the start of germination trials.

### 4.2. Seed Characterisation

**Figure 8:**
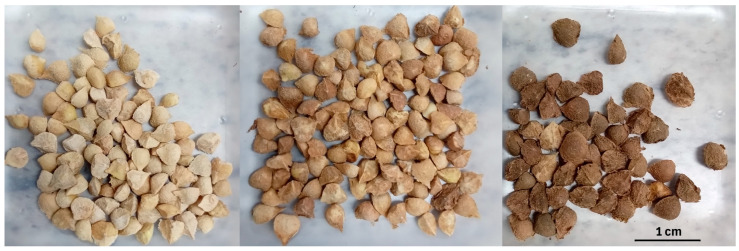
The differently classified seeds of *B. pirottae* were (1) light brown (**left**); (2) brown (**middle**); and (3) dark brown (**right**).

While extracting seeds from the fruits, three distinct seed colours were observed: light brown (LB), brown (B), and dark brown (DB) (Figure 8). Based on this observation, seeds were manually sorted into three categories based on seed colour—LB, B, and DB.

The average number of seeds per fruit was calculated by assessing 100 randomly selected fruits from the bulk collected sample. Then, the fruits were de-pulped, and the number of seeds per fruit was counted and recorded in a data collection sheet independently.

The mean seed mass of each seed colour category (LB, B, and DB) was determined by weighing 100 randomly selected seeds from each seed colour category independently. Individual seeds were weighed using an electronic balance. The number of seeds per kilogram was determined by counting 1000 seeds from the uncategorised batch (not sorted by colour). Finally, the mass of 1000 seeds were measured following the guidelines outlined in ISTA [75].

The buoyancy (floating and sedimenting) characteristics of seeds for each seed colour category (LB, B, and DB) were determined from 60 randomly selected seeds with similar dimensions of each colour category and divided into four replicates (each with 15 seeds). After that, seeds in respective replicates were placed in a cup of water and left submerged for twenty-four hours within a temperature-controlled chamber set at 23 °C. After twenty-four hours, the number of seeds that floated and sank was counted.

To determine the water imbibition rate of different seed colours, 50 seeds from each category (LB, B, and DB) with similar dimensions were randomly selected. The initial masses (dry masses) of the individual seeds were measured for each category using an electronic balance before submersing them into water. The seed samples were then immersed in distilled water, stirred for five minutes, and left to soak further in a chamber maintained at a constant temperature of 23 °C. After soaking for 3 h, the immersed seeds were taken from the chamber and placed on blotting paper to aid in their drying, and individual seeds were weighed using an electronic balance. This process was repeated after 6 h, 24 h, and 27 h of soaking.

### 4.3. Germination Trials

**Figure 9:**
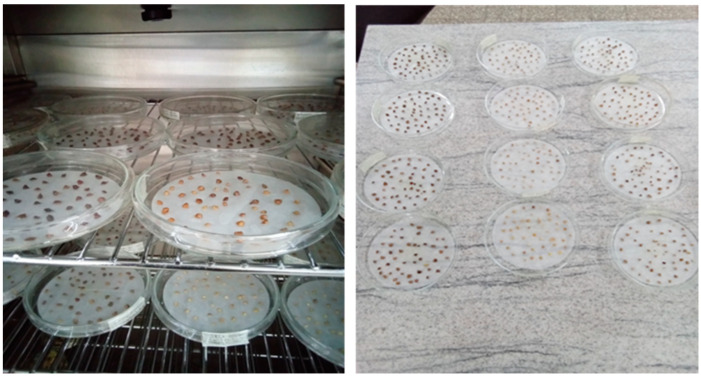
Germination experiment conducted at a controlled temperature in the germination chamber (**left**) and at room temperature (**right**).

The seed germination of each colour seed set of *B. pirottae* (LB, B, and DB) was performed in a seed laboratory. Before starting the germination test, seeds of different colours were independently immersed in water. The different-coloured floating seeds were not used for the experiment, due to the assumption that they might be empty seeds. Therefore, only submerged seeds were used for the germination test, as they were assumed to be viable. Seeds were germinated on a filter paper in a Petri dish (Figure 9). The germination trials were performed in a seed germination chamber with a controlled temperature of 23 °C and at room (fluctuating) temperature (at the minimum temperature of 19 °C in the morning and a maximum temperature of 25 °C in the late afternoon; average daily temperature of 21 °C). The seed laboratory had a single chamber set to a constant 23 °C, and since the species is fire-adapted, we assumed that it requires higher temperatures for optimal seed germination.

A randomised complete block design (RCBD) was used in the germination experiment. For each seed colour category (LB, B, and DB) and temperature, 200 seeds were used independently. These seeds (200 seeds) were divided into four replicates, with 50 seeds in each. Watering was undertaken daily in the morning until the end of the experiment. To prevent fungal growth in the Petri dishes, sown seeds were carefully rinsed with distilled water weekly and transferred to new and cleaned Petri dishes with a new filter paper. Germination data were recorded daily. Seeds were considered germinated when the length of the radicle was at least 2 mm. Ungerminated seeds were dissected at the end of the experiment using a sharp knife. Seeds were visually inspected after being cut, opened with a knife, and classified as either non-viable or viable based on FAO [76] criteria. Seeds were classified as non-viable if they comprised soft, decayed, or malformed embryos, and viable seeds were those with well-formed, normal-coloured endosperms and healthy embryos.

### 4.4. Data Analysis

The mean final germination percentage (GP) was determined using the methodology outlined by Scott et al. [77] (Equation (1)), while the mean germination time (MGT) was calculated based on Orchard’s method [78] (Equation (2)). The mean germination index (GI) was assessed following the method by Bench et al. [79] (Equation (3)). Moreover, the mean water imbibition rate (MWI) of the seeds was evaluated using Equation (4). The mean daily germination analysed following the procedure of Czabator [80] was presented as a peak value, which is defined as the highest quotient obtained by dividing the number of germinates accumulated on a given day by the corresponding number of days. This represents the mean daily germination rate of the most vigorous seeds in the seed lot.

For data analysis, parametric tests were used since the data fulfil the assumption of normal distribution. One-way ANOVA and *t*-tests were used for data analysis. Fisher’s least significant difference test was used for the mean separation at a significance level of 0.05. Sigma Plot 13 (Systat Software, Inc., San Jose, CA, USA) was used for data analysis.

The final germination percentage (GP) was calculated using Equation (1):

GP = TG/TS(1)

where TG denotes the total number of seeds germinated, and

TS denotes the total number of sown seeds.

The mean germination time (MGT) of seeds was calculated using Equation (2):

(2) $$MGT=\frac{\sum_{n=1}^{n}T1N1+T2N2+\ldots +TkNk}{\sum_{n=1}^{n}N1+N2+\ldots +Nk}$$

where N denotes the number of newly germinated seeds, and

T denotes the time from the beginning of the experiment.

The germination index (GI) was calculated using Equation (3):

(3) $$GI=\sum_{n=1}^{n}\left(T1N1+T2N2+\ldots TkNk\right)$$

where N denotes the number of newly germinated seeds, and

T denotes the time from the beginning of the experiment.

The mean water imbibition rate (MWI) was analysed using Equation (4):

(4) $$MWI=\frac{\sum_{n=1}^{n}(W1+W2\ldots .+WK)-(D1+D2\ldots DK)}{\sum_{n=1}^{n}D1+D2+\ldots +Dk}$$

where W denotes the seeds’ wet mass, and

D denotes their dry mass.

## 5. Conclusions

In conclusion, our study showed that the seeds of *B. pirottae* vary with respect to their colour. The results showed that the seed colour and seed mass influence the mean germination of *B. pirottae* seeds. In contrast, the temperature treatment revealed no significant differences with respect to the mean germination of differently coloured seeds that were treated at a constant temperature (23 °C) and at fluctuating room temperature. Overall, our research showed that while there is a need for seedling raising for the conservation and afforestation of *B. pirottae* species, it is recommended to use dark brown seeds because of the relatively higher mean germination percentage compared to light brown and brown seeds.

## Data Availability

Data used in this article are available upon request to the authors.
